# Tenomodulin Expression in the Periodontal Ligament Enhances Cellular Adhesion

**DOI:** 10.1371/journal.pone.0060203

**Published:** 2013-04-10

**Authors:** Yuske Komiyama, Shinsuke Ohba, Nobuyuki Shimohata, Keiji Nakajima, Hironori Hojo, Fumiko Yano, Tsuyoshi Takato, Denitsa Docheva, Chisa Shukunami, Yuji Hiraki, Ung-il Chung

**Affiliations:** 1 Department of Sensory and Motor System Medicine, The University of Tokyo, Bunkyo-ku, Tokyo, Japan; 2 Department of Oral and Maxilofacial Surgery, The University of Tokyo, Bunkyo-ku, Tokyo, Japan; 3 Center for Disease Biology and Integrative Medicine, The University of Tokyo, Bunkyo-ku, Tokyo, Japan; 4 Laboratory for Experimental Surgery and Regenerative Medicine, Department of Surgery, Ludwig-Maximilians-University, Munich, Germany; 5 Department of Cellular Differentiation, Institute for Frontier Medical Sciences, Kyoto University, Kyoto-city, Kyoto, Japan; University of Sydney, Australia

## Abstract

Tenomodulin (Tnmd) is a type II transmembrane protein characteristically expressed in dense connective tissues such as tendons and ligaments. Its expression in the periodontal ligament (PDL) has also been demonstrated, though the timing and function remain unclear. We investigated the expression of Tnmd during murine tooth eruption and explored its biological functions *in vitro*. Tnmd expression was related to the time of eruption when occlusal force was transferred to the teeth and surrounding tissues. Tnmd overexpression enhanced cell adhesion in NIH3T3 and human PDL cells. In addition, *Tnmd*-knockout fibroblasts showed decreased cell adhesion. In the extracellular portions of Tnmd, the BRICHOS domain or CS region was found to be responsible for Tnmd-mediated enhancement of cell adhesion. These results suggest that Tnmd acts on the maturation or maintenance of the PDL by positively regulating cell adhesion via its BRICHOS domain.

## Introduction

The periodontal ligament (PDL) is a supporting tissue that plays important roles in connecting teeth to the jaw, sensing occlusal force, and providing shock absorption [1]. Periodontitis is one of the world’s most common infectious diseases, affecting approximately 40% of adults aged 35–44 [2]. Microorganisms in the oral cavity and an aggressive response of the immune system against them cause this inflammatory disease [3], ultimately resulting in loss of the functions of the PDL and involved teeth. In addition, recent studies have reported a close relation between severe periodontitis and the progression of diseases such as type 2 diabetes mellitus [4], rheumatoid arthritis [5], hypertension [6], [7], and atherosclerosis [8], [9], indicating the importance of oral healthcare. Once the PDL is destroyed, regeneration is challenging; reliable treatment to fully recover the PDL is limited due to the shortage of knowledge about how the PDL is developed and maintained during and after tooth eruption. Therefore, understanding the nature of the PDL in order to establish an effective and practical regeneration strategy is an important task for both medical and dental health care.

Tnmd is a type II transmembrane protein [10], and it has been reported to characteristically express in dense connective tissues such as tendons and ligaments [11], [12]. The structure of Tnmd includes a highly conserved C-terminal domain (CTD), putative enzyme cleavage site (CS), BRICHOS domain that has two glycosylation sites, transmembrane region, and cytoplasmic tail [10], [13], [14]. The CTD contains eight cysteine residues that form four disulfide bonds, which are also conserved in chondromodulin-1 (ChM-1). The CS contains the R-X-R/L-R basic target sequence for furin, an endoprotease enriched in the Golgi apparatus that functions as a proprotein convertase [11], [12], [15], [16]. The BRICHOS domain contains two conserved cysteines that are presumed to form disulfide bonds and two N-glycosylation sites. The framework of this domain has been suggested to be similar to that of the GroEL apical domain, which is the polypeptide recognition domain [17]. Suggested functions of this domain, including targeting of the secretory pathway, may assist the specialized intracellular protease processing system and provide an intramolecular chaperone-like function, although its role in cellular function is unclear [14].

Tnmd expression has been confirmed with RT-PCR and *in situ* hybridization of brain, heart, tendon, ligament, sclera, and cornea [11], [12], [18], [19], [20]. As for its biological function, the CTD of Tnmd has been shown to have the anti-angiogenic effect, when overexpressed in endothelial cells [21]. Given that Tnmd is known to emerge in the late developmental stage of tendons and ligaments [19] and the PDL is categorized as a dense connective tissue, it is possible that Tnmd has roles in the development and maintenance of the PDL and serves as a biomarker characterizing functions of it. However, there has been no report on the expression pattern or biological function of Tnmd in periodontal tissues.

In the present study, we aimed to understand the biological roles of Tnmd in the PDL. We established rabbit polyclonal anti-Tnmd antibodies and investigated the pattern and timing of Tnmd expression in the PDL of wild type (WT) mice in comparison with those of *Tnmd*-knockout (*Tnmd*-KO) mice. We further examined the cellular functions of Tnmd in NIH3T3 cells and human immortalized PDL cells as well as its subcellular localization.

## Materials and Methods

### Cells, Plasmid Vectors, and Mice

NIH3T3 cells were obtained from RIKEN Cell Bank. Human immortalized PDL cells (hPDL-*TERT*) were established as described previously [22]. Cells were cultured in Dulbecco’s modified Eagle medium (DMEM) containing 10% fetal bovine serum (FBS) at 37°C under 5% CO2 in air. pCAGGS-*FLAG-mTnmd* was generated by polymerase chain reaction (PCR) using pCAGGS as described previously [10]. A bicistronic expression vector with *Venus* and *FLAG-Tnmd* was generated using PCR. *Tnmd*-KO mice were generated as described previously [23]. Littermates were mated to generate Tnmd heterozygotes or hemizygotes. Genotypes and sex were identified using PCR with the following primers: Tnmd WT, F:aactccacctcagcagtactcc; Tnmd KO, F:gattagataaatgcctgctc; Tnmd WT/KO, R:ttcttggatacctcgggccag; mSry, F:ctcatcggagggctaaagtg; and mSry, R:aagctttgctggtttttgga. All animal experiments were approved by the animal welfare committee of Tokyo University Graduate School of Medicine.

### Establishment of Anti-Tnmd Specific Antibody

**Figure 1:**
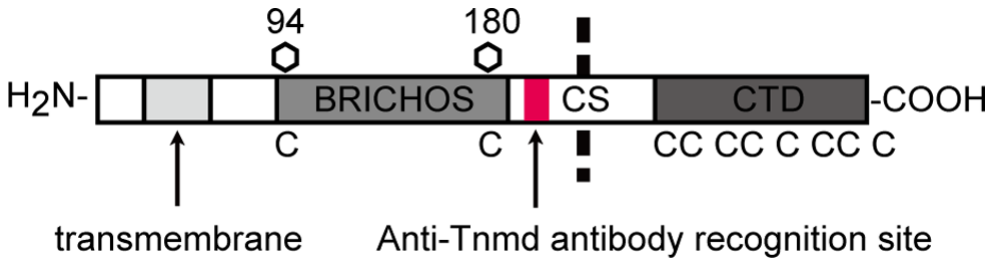
Illustration of Tnmd structure and design of antibody recognition position. Schematic illustration of the Tnmd structure. Domain structures are highlighted. The position of the antibody recognition site is indicated in red. The N-glycan glycosylation site is indicated by a hexangular box with the amino acid number. Conserved cysteine residues are indicated in capital letters. Transmembrane: transmembrane region; BRICHOS: BRICHOS domain; CS: possible cleavage site; CTD: C-terminal domain.

A polyclonal antibody to mouse Tnmd was raised by immunizing rabbits with a polypeptide corresponding to the region with amino acids LHFPTSEKKGIDQNE (Figure 1). Immunization was performed every week for 5 weeks and the obtained serum was titered on week 6. When a sufficient antibody titer was confirmed, the antigen was administered again as a booster. Whole serum samples were collected on week 7 and ProClin (Sigma-Aldrich) was added to preserve the samples until affinity purification at −80°C. For purification, antisera were precipitated with 45% ammonium sulfate and centrifuged. The pellet was dissolved in phosphate buffered saline (PBS) and desalted using a PD-10 column (GE Healthcare), and then the protein solution was loaded onto an affinity column overnight at 4°C on a rotator. The column was then washed with PBS and the antibody eluted using 3 M MgCl. The protein concentration of each fraction was measured using a spectrophotometer (Nanodrop-1000; Thermo Scientific), then collected and desalted again, and buffered with PBS. The antibody solution was concentrated by ultrafiltration (Millipore, Bedford, MA), and equal volumes of glycerol and 15 mM NaN3 were added to be stored at −30°C until use in the experiments.

### Cleavage of N-glycans and Inhibition of Glycosylation *in vitro*

NIT3T3 cells were seeded at 4.0×10^4^ cells/dish and cultured for 10–12 hours. At 48 hours after transfection, cells were collected in 1% SDS and sonicated for 3 minutes on ice. The lysates were centrifuged at 15,000 rpm for 20 minutes at 4°C, and then the supernatants were treated with PNGaseF (Sigma Aldrich, St. Louis, MO) for various amounts of time. Tunicamycin (Sigma-Aldrich) was used to inhibit glycosylation. NIT3T3 cells were seeded at 4.0×10^4^ cells/dish and cultured for 10–12 hours. At 24 hours after transfection, 0.5 or 1.0 µg/ml of tunicamycin was added. The cells were harvested at 48 hours after transfection and subjected to western blot analysis.

### Cell Fractionation, Gels, Coomassie Brilliant Blue (CBB) Staining, and Western Blot Analysis

To obtain the intracellular fraction, a ProteoExtract Subcellular Extraction kit (Calbiochem, La Jolla, CA) was used according to the manufacturer’s instructions. Each fraction was denatured by boiling in sample buffer containing 50 mM DTT for 5 minutes and subjected to SDS-PAGE. For whole cell analysis, cultured cells were washed with PBS twice and fixed with 10% trichloroacetic acid (TCA) on ice for 20 minutes. After fixation, cells were collected in micro-centrifugation tubes and washed twice with PBS. The pellets were eluted in buffer containing 2 M Urea, 7 M Urea, 3% CHAPS, and 1% TritonX-100. Cell lysates were collected by centrifugation at 15,000 rpm for 30 minutes at 4°C, then mixed with 4x SDS sample buffer. Samples were electrophoresed on 10–20% SDS-polyacrylamide gels. For CBB staining, the gel was stained with 50 ml of Bio-safe CBB G-250 (Bio-rad Laboratories Inc., Hercules, CA) for 1 hour and washed overnight with distilled water. The gels were then imaged with a digital photoscanner GT-X700 (Seiko Epson Corp., Nagano, Japan). For western blot analyses, gels were transferred onto Immobilon-P membranes (Millipore, Bedford, MA). After pre-incubation with blocking buffer (1% bovine serum albumin, 0.1% Tween 20, in TBS), the membranes were incubated with either a rabbit anti-Tnmd polyclonal antibody against the synthetic peptide corresponding to the human/mouse Tnmd sequence from Thr124 to Glu136 or anti-FLAG polyclonal antibody (Sigma Aldrich), followed by incubation with horseradish peroxidase (HRP)-conjugated anti-rabbit or anti-mouse IgG antibodies (Promega, Madison, WI). Peroxidase activity was visualized using an enhanced chemiluminescence (ECL) plus system (Amersham Pharmacia Biotech Inc., Buckinghamshire, UK), according to the manufacturer’s instructions.

### Immunocytochemistry

NIH3T3 cells were seeded on cover glasses and cultured for 10–12 hours. For transfection, 0.2 synthetic peptide corresponding to the human/mouse Tnmd sequence from Thr124 to Glu136 or anti- washed twice with PBS and fixed with 1% glutaraldehyde supplemented with MgCl_2_ and saponin for 20 minutes. Each specimen was washed twice with PBS and blocked with Tris-buffered saline containing 1% bovine serum albumin and 0.05% Tween-20 (BSA/TBS-T) for 1 hour at room temperature, then incubated with the following primary antibodies overnight at 4°C: anti-pan-cadherin antibody (anti-PC antibody) at 1∶200, wheat germ agglutinin Tetramethylrhodamine conjugate (WGA) at 1∶1000, anti-beta actin antibody at 1∶400, anti-alpha tubulin antibody at 1∶400, and anti-Tnmd antibody at 1∶400. The specimens were then incubated with the following secondary antibodies for 1 hour at room temperature: anti-mouse IgG antibody Alexafluor 546 conjugate at 1∶1000, and anti-rabbit IgG antibody Alexafluor 546 or 647 conjugate at 1∶1000. The specimens were enclosed with Aquatex (Merck KGaA, Darmstadt, Germany) and stored at 4°C away from light until observations were performed with a confocal laser scan microscope LSM510 (Carl Zeiss Microscopy Co., Ltd., Tokyo, Japan). Obtained digital images were processed using Adobe Photoshop^®^ software (Adobe Systems Inc., Tokyo, Japan).

### Histological Analysis

1, 2, 3, 4, 6, or 10-week-old mice were euthanized with isoflurane inhalation. Before fixation, 30 mM HEPES was perfused to wash out blood, and then 10% neutral buffered formalin was used for perfusion fixation and the tissue of interest was isolated. Tissues were further fixed overnight at 4°C and decalcification was performed with 20% EDTA (pH 7.4) at 4°C for 7–10 days, with the solution changed every day. The tissues were then washed with water and embedded in paraffin. Sectioning was done using a standard technique to produce 4-µm thick sections that were utilized for further analysis. The specimens underwent Hematoxylin and eosin (H&E) staining according to a standard protocol. Images were taken by an Axio imager.A1 microscope (Zeiss) as described below utilizing a x2.5 apochromatic objective lens. Digital images were captured by Axiocam MRc and Axiovision ver. 4.6 (Zeiss) and processed using Adobe Photoshop^®^ software (Adobe Systems Inc.). The 10-week-old mouse specimens were subjected to microfocus X-ray CT analysis by using an inspeXio SMX-90CT (Shimadzu Corporation, Kyoto, Japan) according to the manufacturer’s instructions.

### Immunohistochemistry

The specimens were deparaffinized and immersed in water, followed by an antigen retrieval procedure with a 0.05% Trypsin-EDTA solution for 15 minutes at room temperature. Each specimen was neutralized in TBS and blocked with 1% BSA/TBS-T for 1 hour, and then endogenous peroxidase was inactivated using 0.3% H_2_O_2_. Anti-Tnmd antibody was used as the primary antibody at 1∶400 and cultured overnight at 4°C. For the secondary antibody, MAX-PO rabbit IgG (Nichirei, Tokyo, Japan) was added for 1 hour at room temperature. Each specimen was washed with TBS-T and visualized with AEC (Nichirei), then counterstained with hematoxylin. Enclosed specimens were observed under a light field microscope, Axio imager.A1 (Zeiss), utilizing a x2.5, x5, or x10 apochromatic objective lens. Digital images captured by Axiocam MRc and Axiovision ver.4.6 (Zeiss) were processed using Adobe Photoshop^®^ software (Adobe Systems Inc.).

### Cell Adhesion Assay

Ninety-six-well microtiter plates were coated with 1% BSA, a type I collagen (Col I) solution and incubated overnight at 4°C. To prevent the cells from directly attaching to the microtiter plates, 1% BSA was used. The coated plates were dried at room temperature and washed twice with sterile PBS, and then cells transfected with expression vectors were seeded on the plates. After 1 hour or 30 minutes of incubation for NIH3T3 or hPDL-*TERT* cells, respectively, the input fluorescence of each well was detected using a Typhoon confocal laser scanner (GE Healthcare, Buckinghamshire, UK) or InCell analyzer 1000 (GE Healthcare). The plates were then washed with PBS 3 times and the fluorescence of each well was detected again as the adherent cell fluorescence. The entire fluorescence value for each well in terms of cell numbers was measured using the Typhoon scanner and the adherent cell ratio was calculated.

### RNA Isolation, Reverse Transcription, and Quantitative Polymerase Chain Reaction (qPCR)

Total RNA was isolated using an ISOGEN kit (Wako Pure Chemical Industries, Ltd., Tokyo, Japan) and treated with DNase 1 (Qiagen, Hilden, Germany), following the manufacturer’s instructions. After reverse-transcription using a QuantiTect^®^ Reverse Transcription Kit (Qiagen), PCR was performed with an ABI Prism 7000 Sequence Detection System (Applied Biosystems, Foster City, CA) using QuantiTect SYBR Green PCR Master Mix (Qiagen). All reactions were run in triplicate. The mRNA copy number of a specific gene was calculated with a standard curve generated using serially diluted plasmids containing PCR amplicon sequences, and normalized to human or rodent total RNA with mouse actin used as the internal control. Standard plasmids were synthesized using a TOPO TA cloning Kit (Invitrogen, Carlsbad, CA), according to the manufacturer’s instructions. Data are expressed as the means±SD. The primer sequences are available upon request.

### Statistical Analysis

The significance of differences was determined with Student’s t-test in the results of cell adhesion assay. The mean values of groups were compared using ANOVA and the significance of differences was determined with Tukey’s post hoc test in the results of cell adhesion assay for deletion mutants.

## Results

### Establishment of Anti-Tnmd Antibody

To explore the expression pattern of Tnmd in mouse PDLs, we established a polyclonal antibody against an amino acid sequence of the N-terminal side of the CS region of mouse Tnmd (Figure 1). In western blot analysis of NIH3T3 cells transfected with *Tnmd* (*Tnmd*-overexpressing NIH3T3 cells), the anti-Tnmd antibody detected 45-and 40-kDa bands (Figure S1A), which was in agreement with previous reports [10], [15], [20], [24]. Anti-Tnmd antibody did not recognize any proteins in NIH3T3 cells transfected with or without the unrelated GFP gene. We also examined FLAG-tagged Tnmd (FL-Tnmd) using an anti-FLAG antibody to compare with the results obtained with the anti-Tnmd antibody. In NIH 3T3 cells transfected with FLAG tagged-*Tnmd* (*FL-Tnmd*-overexpressing NIH3T3 cells), the anti-FLAG antibody detected 45- and 40-kDa bands, which were the same as those detected with anti-Tnmd antibody on the same membrane (Figure S1B). To further examine the specificity of the anti-Tnmd antibody, immunohistochemistry (IHC) was performed with tail tissues of *Tnmd*-KO and WT mice (Figure S1C). Normal rabbit serum was used as a negative control, and did not detect any signal in either strain of mice. Anti-Tnmd antibody detected a specific signal in tenocytes of the WT mice, with higher staining intensity in the insertion of tendon, while no signal was detected in the *Tnmd*-KO mice (Figure S1C). Taken together, these results showed that the anti-Tnmd antibody was able to specifically detect Tnmd in both western blot analysis and IHC.

### Analysis of Tnmd Expression in the PDL

**Figure 2:**
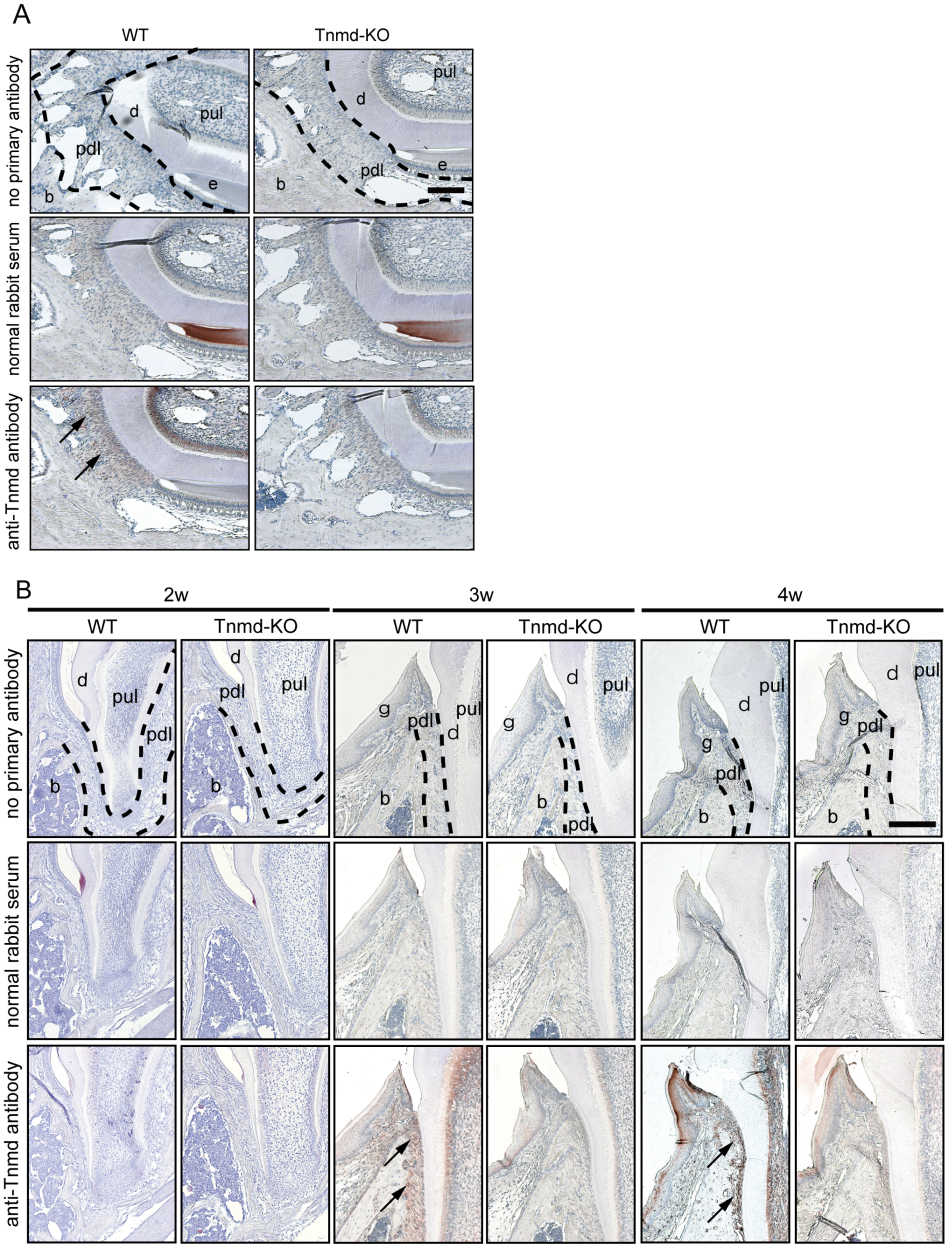
Expression of Tnmd in the mouse periodontal ligament. (**A**) Immunohistochemical detection of Tnmd in the incisors. Tnmd localization was examined by IHC in mandible incisors obtained from 4-week-old mice. (**B**) Immunohistochemical detection of Tnmd in molars. The temporal expression of Tnmd was examined by IHC in maxilla molars obtained from 2, 3, and 4-week-old mice. e: enamel organ; d: dentin; g: gingiva; pul: pulp; pdl: periodontal ligament; b: bone. The dotted line indicates the area of periodontal ligament. Arrow indicates the region of positive signal. Scale bar = 500 µm.

Murine incisors continue to erupt throughout life, while murine molars stop erupting with the formation of an apex of the root at some point in life. Hence, it is considered that the morphology, mechanism of root formation, and pattern of eruption in murine molars were similar to those in humans. H&E staining of 1, 2, 3, 4, and 6-week-old WT mouse molars revealed that their eruption was likely to occur between the ages of 2 and 3 weeks (Figure S2). Two-week-old mouse molars were still covered by oral mucosa. In contrast, the crown tip had already penetrated the oral mucosa in 3-week-old mice (Figure S2). Attrition of the crown tip was observed in 4-week-old mice, suggesting that the molars had started to function. Taken together, these results showed that molars in the 2, 3, and 4-week-old mice were likely in the preeruptive, eruptive, and posteruptive phase, respectively. Therefore, we performed IHC of Tnmd to investigate expression in 4-week-old mice incisors and molar PDLs, i.e., in the posteruptive phase. IHC without the primary antibody or with normal rabbit serum served as a negative control, which did not detect positive signals from any of the tested specimens. In contrast, IHC of Tnmd revealed positive signals in PDL cells, odontoblasts, and periosteum cells in 4-week-old WT mouse incisor and molar specimens, while those in *Tnmd*-KO mice did not have such signals (Figure 2A and B). Positive signals were also observed in 3-week-old WT mouse molars. Such signals were barely observed in 2-week-old WT mouse molars (Figure 2B). Although Tnmd expression in the PDL was detected in the eruptive and posteruptive phases, the molar eruption in *Tnmd*-KO mice was not delayed.

To explore the function of Tnmd in the PDL, craniocervical hard tissues from WT and *Tnmd*-KO mice were analyzed using a microfocus X-ray CT system. There were no gross abnormalities in tooth number, number of tooth roots, or morphology of the craniocervical hard tissues, including the teeth, cranial bones, facial bones, and jaw bones (Figure S3). Thus, Tnmd does not affect hard tissue development in the oral and craniofacial regions.

### Subcellular Localization of Tnmd

**Figure 3:**
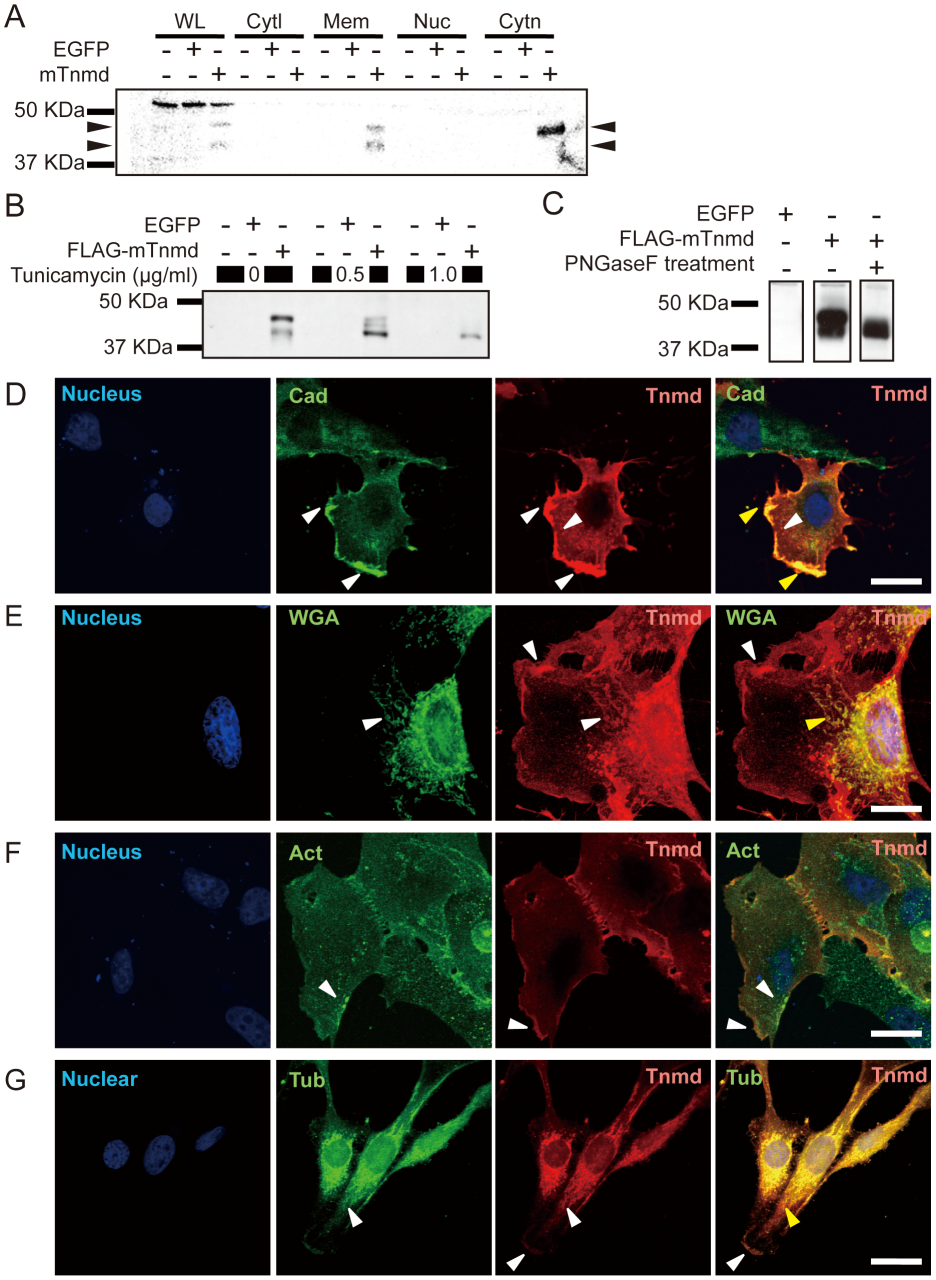
Determination of the subcellular localization of Tnmd. (**A**) Detection of Tnmd in cell fractions by western blotting. (**B**) Examination of the glycosylation of Tnmd with tunicamycin treatment. (**C**) Examination of the N-glycosylation of Tnmd with PNGase treatment. WL, whole cell lysate; Cytl: cytosol fraction; Mem: membrane fraction; Nuc: nuclear fraction; Cytn: cytoskeleton fraction. (**D-G**) The subcellular localization of the Tnmd protein in NIH3T3 cells transfected with Tnmd was examined by ICC. NIH3T3 cells were stained with cell organelle markers to indicate the plasma membrane [upper panels]. NIH3T3 cells transfected with Tnmd were double stained with cell organelle markers and the anti-Tnmd antibody [lower panels]. (**D**) Tnmd and plasma membrane marker, Pan-Cadherin (Cad). (**E**) Tnmd and Golgi apparatus marker, Wheat germ agglutinin (WGA). (**F**) Tnmd and β-Actin (Act). (**G**) Tnmd and α-Tubulin (Tub). SYTOX nuclear staining is shown in blue, cell organelle markers in green, and anti-Tnmd antibody in red. White arrowhead indicates the positive staining or organelle markers or anti-Tnmd antibody. Yellow arrowhead indicates the colocalization of the organelle marker signal and Tnmd signal. Scale bar = 50 µm.

The subcellular localization of Tnmd was investigated in *Tnmd*-overexpressing NIH3T3 cells by western blot analysis of fractionated lysates. Each fraction showed unique band patterns in CBB staining of SDS-PAGE gels, which supported the integrity of cell fractionation (data not shown). In western blot analysis, anti-Tnmd antibody detected 45- and 40-kDa protein signals in the whole cell lysates and membrane fractions, and a 45-kDa protein signal in the cytoskeleton fraction (Figure 3A, Figure S4). Since the two different signals of Tnmd have been reported to cause by N-glycosylation [15], we examined the N-glycosylation of Tnmd by adding tunicamycin, an inhibitor of glycosylation, to the culture (Figure 3B) and by digesting the cell lysates with PNGaseF (Figure 3C). Cells treated with 0.5 mg/ml tunicamycin showed signals for a 45, 42.5, and 40-kDa protein, and those treated with 1.0 mg/ml tunicamycin showed a signal for a 40-kDa protein. Treatment of cell lysates with PNGaseF also eliminated the 45-kDa protein. Thus, we considered that the 45-kDa protein was likely glycosylated Tnmd and the 40-kDa protein was likely non-glycosylated Tnmd.

We next performed immunocytochemistry (ICC) of *Tnmd*-overexpressing NIH3T3 cells. Tnmd was colocalized with the cell membrane marker Pan-cadherin (Figure 3D) as well as the Golgi apparatus marker WGA (Figure 3E). When co-stained with the cytoskeleton markers, Tnmd was colocalized with actin (Figure 3F). Tubulin also tended to be colocalized with Tnmd (Figure 3G), though Tnmd staining was richer in the membrane. Thus, the protein was localized in the Golgi apparatus, cytoskeleton, and cell membrane.

### Morphological Analysis of Tnmd-transfected NIH3T3 Cells

**Figure 4:**
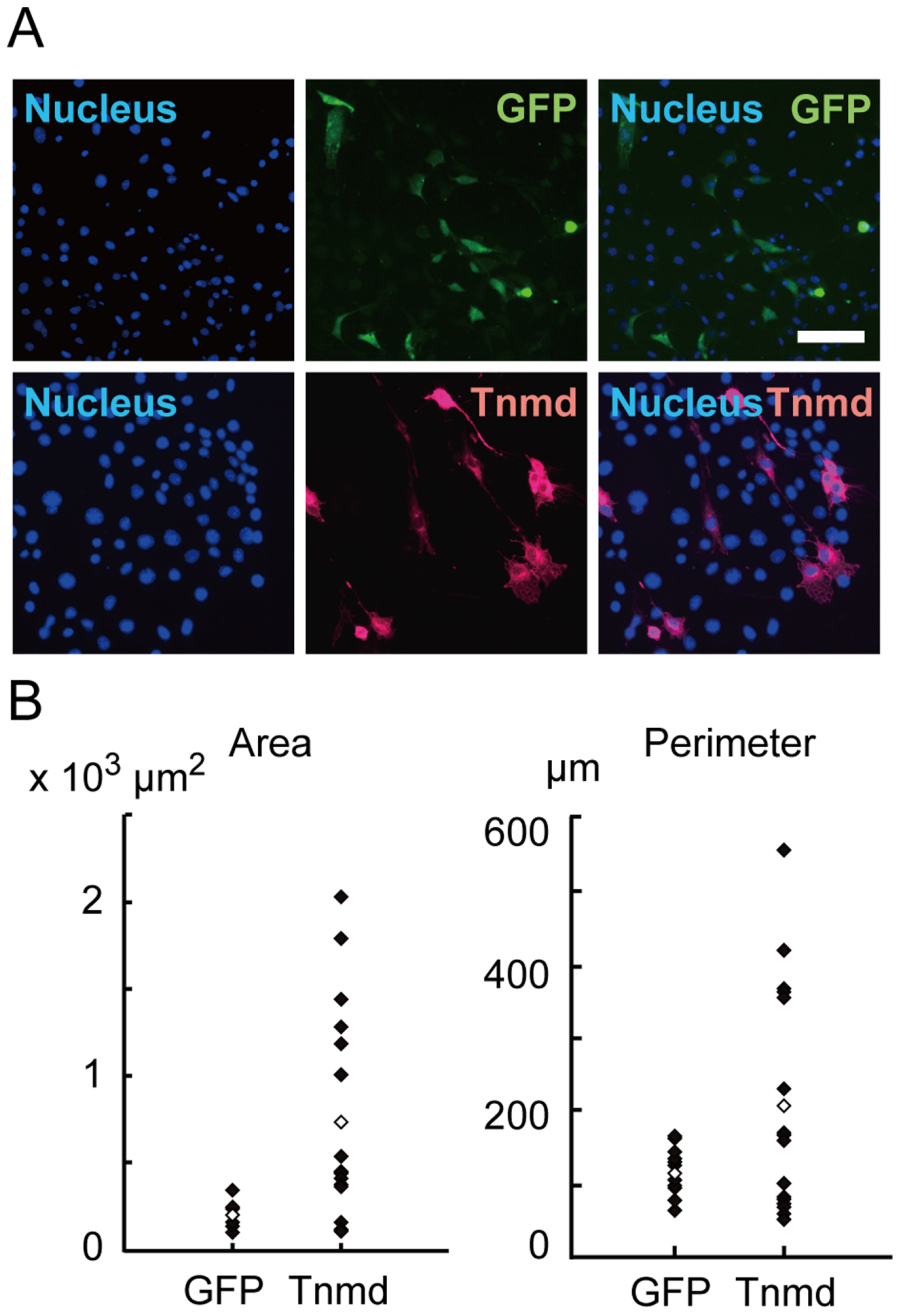
Morphological changes of Tnmd-transfected NIH3T3 cells. (**A**) Immunocytochemical detection of Tnmd in NIH3T3 cells transfected with *Tnmd* or *GFP*. DAPI: nuclear staining. Scale bar = 50 µm. (**B**) Measurement of the cell area and perimeter of NIH3T3 cells transfected with *Tnmd* or *GFP*. Cells in 5 microscopic fields were measured. Tnmd group: n = 16; GFP group: n = 13. White spot: average value.

Since *Tnmd*-overexpressing NIH3T3 cells tended to have longer cell processes than NIH3T3 cells transfected with *GFP*, as shown by microscopic observations (Figure 4A), we compared the morphology of *Tnmd*-overexpressing NIH3T3 cells with that of *GFP*-overexpressing NIH3T3 cells by measuring the area and cell perimeter (Figure 4B). Both parameters were significantly increased in Tnmd-overexpressing NIH3T3 cells as compared to *GFP*-overexpressing NIH3T3 cells (743.3±607.7 µm^2^ vs. 207.6±59.0 µm^2^, p = 0.005; 207.1±151.6 µm vs. 115.8±31.0 µm, p = 0.049). These results indicate that Tnmd may influence cell morphology.

### Effect of FLAG-tagged Tnmd Transfection on Adhesion

**Figure 5:**
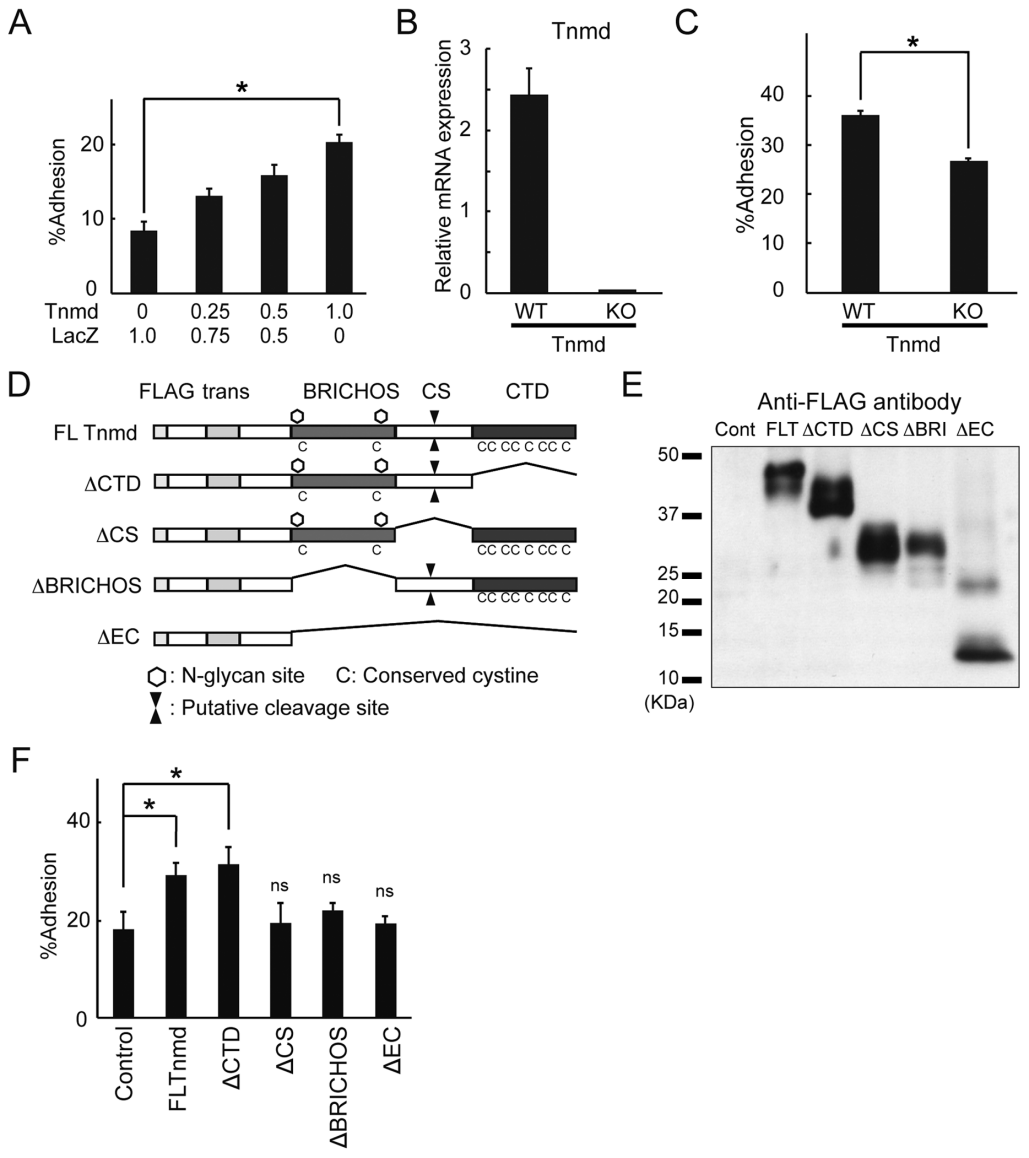
Effect of Tnmd transfection on cell adhesion of fibroblastic cells. (**A**) Dose-dependent enhancement of cell adhesion by Tnmd. FLAG-tagged *Tnmd* was transfected in combination with pCAGGS-Venus in NIH3T3 cells. Cell adhesion to col I-coated culture dishes was determined. The fluorescence intensity was measured using a confocal laser scanner, and the percent cell adhesion was calculated. (**B**) Relative expression levels of *Tnmd* mRNA in WT and Tnmd-KO cells determined by real-time RT-PCR. (**C**) Cell adhesion of WT and *Tnmd*-KO cells to col I-coated culture dishes. (**D**) Illustration of the domain deletion mutants of *Tnmd*. Tnmd FC: FLAG-tagged full length Tnmd; Tnmd ΔCTD (ΔCTD): C-terminal domain deletion mutant; Tnmd ΔCS (ΔCS): mutant with a deleted putative cleavage site; Tnmd ΔBRICHOS (ΔBRI): BRICHOS domain deletion mutant; Tnmd ΔEC (ΔEC): mutant with the entire extracellular portion of the molecule deleted. (**E**) Expression of Tnmd mutants. Expressions of Tnmd mutants were analyzed by western blotting using the anti-FLAG antibody. Each mutant was transfected into NIH3T3 cells. (**F**) Effect of FLAG-tagged Tnmd or domain deletion mutants on cell adhesion in NIH3T3 cells. NIH3T3 cells were transfected with beta galactosidase [control], FLAG-Tnmd, ΔCTD, ΔCS, ΔBRI, or ΔEC in combination with pCAGGS Venus. Cell adhesion to col I-coated culture dishes was determined. The percent cell adhesion was calculated based on the fluorescence intensity measured by a confocal laser scanner. Three measurements were performed for each and representative values are shown as the means±SD. *p<0.05; ns: not significant.

Given that *Tnmd*-overexpressing NIH3T3 cells showed morphological change, we hypothesized that cell adhesion was altered by the introduction of *Tnmd*. To investigate the effect of Tnmd on cell adhesion, we performed a cell adhesion assay, in which cells co-transfected with *Tnmd* and the marker gene were grown on Col I or Fibronectin (FN)-coated culture plates, followed by washout of unattached cells to detect adherent ones. Col I and FN were used for the adhesion assay because it is the major extracellular matrix protein in the PDL. The adhesion ratio of *Tnmd*-transfected cells was significantly increased as compared to that of the beta-galactosidase (LacZ)-transfected controls both to Col I and FN (Figure 5A, Figure S5A), in a transgene-dose dependent manner (p = 0.00013 and p = 0.00024, respectively). This result suggests that Tnmd participates in cell adhesion. The effect of Tnmd on cell adhesion was confirmed using cells isolated from neonatal connective tissues of WT and *Tnmd*-KO mice. The expression of *Tnmd* was confirmed by quantitative RT-PCR (Figure 5B). *Tnmd*-KO cells showed decreased adhesion to both Col I and FN as compared to WT cells (Figure 5C, Figure S5B). To determine which domain was responsible for the effect of Tnmd on cell adhesion, we tested its domain deletion mutants (Figure 5D). The integrity of the WT and mutants was supported by the following two observations. First, each type of mutant showed cellular protein expression comparable to that of the WT (Figure 5E). Second, the transfection efficiency did not differ among them (data not shown). *FLAG-Tnmd*-overexpressing NIH3T3 cells and ΔCTD-overexpressing NIH3T3 cells showed markedly greater adherence to Col I-coated plates compared to the control cells. In contrast, ΔCS-, ΔBRICHOS-, and ΔEC-transfected NIH3T3 cells did not enhance the adherence (Figure 5F). Taken together, these results showed that the CS region and BRICHOS domain in Tnmd may have an important role in cell adhesion to the extracellular matrix.

**Figure 6:**
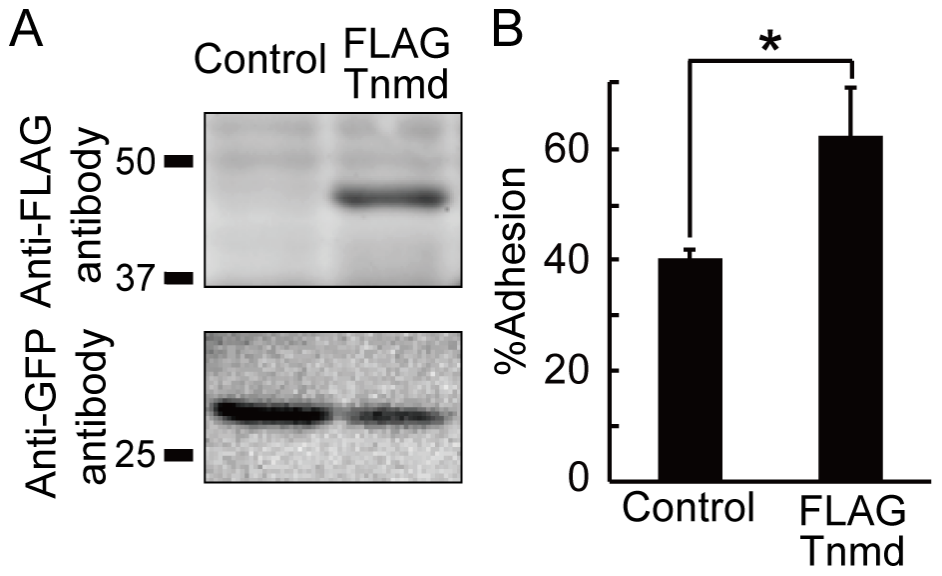
Effect of Tnmd transfection on cell adhesion of PDL-h*TERT* cells. (**A**) Expression of Tnmd in PDL-h*TERT* cells transfected with a bicistronic vector carrying FLAG-tagged *Tnmd* or LacZ and Venus [pEBMulti-FLAG-Tnmd-2a-Venus, pEBMulti-LacZ-2a-Venus], as determined by western blotting. FLAG-tagged Tnmd was detected by anti-FLAG antibody, and Venus was detected by anti-GFP antibody. (**B**) Effect of Tnmd on cell adhesion of PDL-h*TERT* cells to col I-coated culture dishes. The fluorescence intensity was measured using a confocal laser scanner and the percent cell adhesion was calculated.

We also investigated the effect of Tnmd on the adhesion of hPDL-*TERT* cells that were established from human PDL. To observe the adhered cells more accurately, we constructed a bicistronic expression vector expressing both the FLAG-tagged *Tnmd* and *Venus,* an enhanced yellow fluorescent protein, using a 2a peptide sequence. The protein expression and cleavage by the 2a peptide sequence were confirmed on western blot analysis (Figure 6A). *FLAG*-tagged *Tnmd* and *Venus* were detected as their estimated molecular weight, suggesting that the protein was cleaved by the 2a peptide sequence. hPDL-*TERT* cells transfected with the *Tnmd* showed enhanced cell adhesion (Figure 6B). As seen in NIH3T3 cells, Tnmd was localized in the Golgi apparatus, cytoskeleton, and cell membrane in the PDL-hTERT cells (Figure S6). These results suggest that Tnmd participates in the adhesion of PDL cells to the extracellular matrix.

## Discussion

This study had five major findings. 1) Tnmd was modified by two N-glycans, and its CTD was not cleaved in NIH3T3. 2) The Tnmd protein was expressed in the PDL during the eruptive phase in murine molars. 3) Tnmd was localized in the plasma membrane, and transfection of *Tnmd* enlarged the cell territory. 4) Transfection of *Tnmd* enhanced cell adhesion, while loss of *Tnmd* suppressed it. 5) The BRICHOS domain and the CS region were important for the enhancement. Based on these findings, we propose that Tnmd is expressed after the tooth erupts to the oral cavity, and is involved in maturation and maintenance of the structure of the PDL by positively regulating adhesion of PDL cells to ECM.

In western blot analysis, Tnmd was detected as two protein bands in NIH3T3 cells. We investigated whether or not this difference of molecular weight was caused by CTD cleavage or by post-transcriptional modification. We found a change in molecular size from 45 to 40 kDa upon PNGase F treatment of whole cell lysates, which is consistent with a previous report [15]. The cleaved Tnmd is supposed to form two fragments, the larger one including the transmembrane region and BRICHOS domain, and the shorter one corresponding to CTD [24]; the CTD was detected in the tendon [23] and tendious cord of the heart [24] as an approximately 16-kDa protein. If the CTD is released from Tnmd, the antibody utilized in our study should detect the rest of the protein as approximately 29-kDa protein. However, we did not detect the 29-kDa protein. Although this discrepancy might reflect the difference between the *in vivo* and *in vitro* setting, these data suggest that the 45 and 40 kDa molecular size of Tnmd was caused by N-glycosylation, not by protein cleavage. As previously noted by others, Tnmd has two N-glycosylation sequences. The appearance of 3 bands by the addition of 0.5 µg/ml tunicamycin also indicates that both N-glycosylation sites were biologically modified. Glycosylation of protein may play multiple roles, such as modification and maintenance of protein folding, which could directly modulate the function of the protein [25]. Given that the BRICHOS domain was found to be important for the function of Tnmd in this study, N-glycosylation to the BRICHOS domain might be important for the function of Tnmd.

We found that Tnmd expression in the PDL was related to tooth eruption, which occurred at the time when the dental follicle developed into the PDL. The specificity of our anti-Tnmd antibody was investigated on murine tail tendon. The anti-Tnmd antibody detected signals in tendon [11], [12], [26]. We examined the Tnmd expression in the PDL using 2- to 4-week-old mice, in which eruption of the molar teeth occurred, for the following two reasons. First, Tnmd expression is known to increase at the later stage of development of dense connective tissues such as the limb tendon [19]. Second, the PDL began to form shortly after root formation, which occurs at around 2 weeks of age [27]; this age was considered to be at the earlier stage of dense connective tissue formation. In the expression analysis, we attempted to associate Tnmd expression with the three phases of tooth eruption described by Nanci et al. [28]: Tnmd emerged in the PDL at the eruptive phase; its expression became more obvious at the eruptive phase; and its expression was persistent even at the posteruptive phase. In addition, the increase of Tnmd expression in the PDL also appeared to be synchronized with dental attrition, a sign that the occlusal force had been received. These results suggest that the Tnmd expression is linked to the time when the murine teeth began to function.

The localization of Tnmd in the plasma membrane suggests that its function is related to cell adhesion and the control of cell morphology. By cell fractionation, glycosylated and non-glycosylated form of Tnmd was detected in the membrane fraction. Glycosylated Tnmd was detected in the cytoskeletal fraction on western blot. In ICC, Tnmd was colocalized in the Golgi apparatus, microtubules, and plasma membrane. These results suggest that Tnmd that is modified in the Golgi apparatus is transported to the plasma membrane through the intracellular traffic system. Moreover, Tnmd is expressed in odontoblast, a unique cell population that aligns as a single layer between the dentin and pulp and forms dentin throughout life [28]. These cells contribute to the dentin-pulp complex via their cellular processes remaining in formed dentin [29]. The Tnmd expression in odontoblasts may support our findings on the Tnmd-mediated extension of cellular processes.

Our findings on cell adhesion suggest that Tnmd may play a role in cell adhesion to the extracellular matrix. Gain of function of Tnmd enhanced cell adhesion to col I, while loss of Tnmd decreased cell adhesion. Moreover, transfection of Tnmd to hPDL-*TERT* enhanced its adhesion. Despite the enhanced cell adhesion by Tnmd as well as the expression of Tnmd in the PDL, Tnmd does not affect hard tissue development or morphology in the oral and craniofacial regions. We infer that functionally redundant molecules may obscure the phenotype of *Tnmd*-KO mice by compensating for Tnmd in the PDL. Previously, an abnormal ultrastructure of collagen fibril, i.e., an increase in its diameter, was observed in *Tnmd*-KO mice [23]. In the same study, the authors found by IHC of type VI collagen alpha 3 (ColVIα3) that the staining signal was diminished in *Tnmd*-KO mice, while it was normally processed in the WT mice [23]. ColVI lies between the cell surface and collagen bundle, providing an anchoring network for cells to attach through surface receptors in various interstitial tissues [30] and may indirectly regulate the collagen fibrillogenesis [31], [32]. Taken together, these results suggest that Tnmd may enhance the cell adhesion of fibroblastic cells to Col VI.

Recently, Qi J et al. proposed that Tnmd had three isoforms in human tendon tissue [33], raising the possibility that isoform 1, 2, and 3 fulfilled distinct intracellular functions similar to cytosine-specific methyltransferase, SUMO protease, and a-syntriohin, respectively. Given that *Tnmd* is a novel class of protein, it is important to perform studies clarifying the isoforms as well as their structures and functions. With regards to *Tnmd* isoform expression in *Tnmd*-KO, the following data are known, which suggests that isoforms are unlikely expressed in dense connective tissues in mice; northern blot analysis detected no additional mRNA in WT or *Tnmd*-KO mouse [11], [12], [23]; western blot analysis of WT or *Tnmd*-KO mouse tissue samples detected no additional protein with antibody against the C-terminal of Tnmd, which should be able to detect isoform 1 and 3 [23]. It is possible that there are differences between human and mouse and that species-specific isoforms exist. However, information to conclude the mechanisms of Tnmd function, including both the proposed isoform function and enhancement of cell adhesion, is limited. Further comprehensive studies on Tnmd-interacting proteins and its involvement in signal transduction will further clarify mechanisms underlying diverse biological functions of Tnmd, which may give us clues to understand how dense connective tissue maturates. In addition, validation on possible isoforms by northern blot analysis and detection of the protein is necessary. Given the function of Tnmd in cell adhesion and the ultrastructural phenotype of knockouts, Tnmd may contribute to ECM remodeling by enhancing fibroblast adhesion to ECM. Although the deletion of *Tnmd* does not seem to largely affect the morphology of skeletal tissues, it is possible that the elasticity and mechanical properties of the PDL may be affected in Tnmd-KO mice. We may be able to observe such phenotypes, if any, by directly measuring the mechanical property of PDL or controlling the mechanical load to the teeth in aged Tnmd-KO mice. As a model for measuring the mechanical properties of PDL, a tension test might be the most suitable. However, it is technically difficult to remove the tissue required for the testing, due to the complexity of the murine molar morphology. An alternative method for estimating the mechanical properties of PDL would be an occlusal trauma model comparing the periodontal damage between wild types and knockouts. We may be able to observe such phenotypes, if any, by directly measuring the mechanical property of PDL or controlling the mechanical load to the teeth in aged *Tnmd*-KO mice.

The BRICHOS domain and CS region of Tnmd was responsible for Tnmd-mediated enhancement of cell adhesion, indicating that the domain likely contains an effector for the attachment of the cells. The BRICHOS domain has been suggested to have several functions. These include targeting of the secretory pathway, assistance to the specialized intracellular protease processing system, and an intramolecular chaperone-like function [14], [17]. Although some of the members containing a BRICHOS domain have been related to specific diseases, their detailed roles have not been elucidated. Recently, Willander et al. reported the high-resolution structure of the BRICHOS domain of surfactant protein C and indicated that the domain had a protein binding capacity [34]. Considering these results together, we infer that Tnmd interacts with other proteins through the BRICHOS domain to regulate cell adhesion. However, its counterpart and/or whether it may directly or indirectly interact with the ECM are still unclear. We previously showed that the CTD of the Tnmd had an anti-angiogenic activity [10], [20], which indicate that the CTD could be a suppressor of cell-cell interaction or cell-matrix adhesion for endothelial cells. Given that dense connective tissues are hypovascularized, anti-angiogenic activity of the CTD and the function of CS region and/or BRICHOS domain in cell adhesion may cooperatively maintain the characteristics of the tissues including tendons, ligaments, and the PDL.

In conclusion, Tnmd may be involved in cell adhesion, which may be important to maintain collagen fibrils in the periodontal ligament, when the tooth starts to function. Although the trigger of Tnmd expression in the PDL and the detailed function in collagen fibril formation are still unclear, our study shows the relation of Tnmd expression to the time of PDL development. The Tnmd-mediated enhancement of cell adhesion to ECM may be involved in the formation and maintenance of the PDL as well as other dense connective tissues. Further study would reveal the role of Tnmd in dense connective tissue, which may enable us to control the remodeling of dense connective tissue and shed light on the regeneration of the PDL.

## Supporting Information

Figure S1 **Establishment of anti-Tnmd antibody.** (**A**) Detection of Tnmd transfected in NIH3T3 cells. Human Tnmd was transfected in NIH3T3 cells to evaluate antibody reactivity. (**B**) Detection of FLAG-tagged Tnmd transfected in NIH3T3 cells by western blot analysis. Cell lysates of transfected cells were first detected using the anti-FLAG M2 antibody, then subsequently with the anti-Tnmd antibody. (**C**) Immunohistological evaluation of anti-Tnmd antibody. WT and *Tnmd*-KO mice tail specimens were stained without the primary antibody, with normal rabbit serum, or with anti-Tnmd antibody. Arrow indicates the region of positive signal. c: cartilage, hf, hair follicle, m: muscle, n: nerve, t: tendon. Scale bar = 500 µm.
(PDF)

Figure S2 **Histological findings of** ***Tnmd*** **–KO mouse teeth.** H&E stainings of the maxilla first molar of 1, 2, 3, 4, and 6-week-old WT or *Tnmd*-KO mice are shown. Scale bar = 500 µm. Representative images are shown.
(PDF)

Figure S3 **Micro CT analysis of craniofacial hard tissue in 10-week-old WT and** ***Tnmd*** **-KO mice.** (**A**) Sagittal plane section image of WT and *Tnmd*-KO mice. Images are focused on a maxillary incisor, mandibular incisor or molar. (**B**) Frontal plane section image of WT and *Tnmd*-KO mice. Images are focused on a first (M1), second (M2), or third molar (M3) and on the temporomandibular joint (TMJ). (**C**) Horizontal plane section image of WT and *Tnmd*-KO mice. Images are focused on the plane, which intersects the molar root and external auditory canal. mxi: maxillary incisor; mdi: mandibular incisor; mx: maxilla; md: mandibule; tmj: temporomandibular joint; mxm: maxillary molar; mdm: mandibularmolar; eac: external auditory canal.
(PDF)

Figure S4 **Examination of glycosylation in Tnmd by Tunicamycin treatment.** Uncropped image of Figure 3B is shown. Full length Tnmd or EGFP is transfected to NIH3T3 cells. Tunicamycin was added 24 h after transfection, and cells were harvested at 48 h after transfection. No protein was detected lower than 37 kDa or higher than 50 kDa molecular weight.
(PDF)

Figure S5 **Effect of Tnmd of Tnmd mutant transfection on cell adhesion.** Cell adhesion assay to Fibronectin are shown. (**A**) Dose-dependent enhancement of cell adhesion by Tnmd. FLAG-tagged *Tnmd* was transfected in combination with pCAGGS-Venus in NIH3T3 cells. Cell adhesion to Fibronectin-coated culture dishes was determined. (**B**) Cell adhesion of WT and *Tnmd*-KO cells to Col I-coated culture dishes.
(PDF)

Figure S6 **Subcellular localization of Tnmd in hPDL-TERT cells.** The subcellular localization of the Tnmd protein in hPDL-TERT cells transfected with Tnmd was examined by ICC. NIH3T3 cells transfected with Tnmd were double stained with cell organelle markers and the anti-Tnmd . (**A**) Tnmd and plasma membrane. (**B**) Tnmd and Golgi apparatus. (**C**) Tnmd and β-Actin. (**D**) Tnmd and α-Tubulin. DAPI nuclear staining is shown in blue, cell organelle markers in green, and anti-Tnmd antibody in red. Scale bar = 50 µm.
(PDF)
