# Supplementary material for: Tenomodulin Expression in the Periodontal Ligament Enhances Cellular Adhesion
Source: PLoS One. 2013 Apr 10;8(4):e60203. doi: 10.1371/journal.pone.0060203 (PMC3622668; doi:10.1371/journal.pone.0060203)
Supplement: Figure S1 — Establishment of anti-Tnmd antibody. (A) Detection of Tnmd transfected in NIH3T3 cells. Human Tnmd was transfected in NIH3T3 cells to evaluate antibody reactivity. (B) Detection of FLAG-tagged Tnmd transfected in NIH3T3 cells by western blot analysis. Cell lysates of transfected cells were first detected using the anti-FLAG M2 antibody, then subsequently with the anti-Tnmd antibody. (C) Immunohistological evaluation of anti-Tnmd antibody. WT and Tnmd-KO mice tail specimens were stained without the primary antibody, with normal rabbit serum, or with anti-Tnmd antibody. Arrow indicates the region of positive signal. c: cartilage, hf, hair follicle, m: muscle, n: nerve, t: tendon. Scale bar = 500 µm. (PDF) [file pone.0060203.s001.pdf]

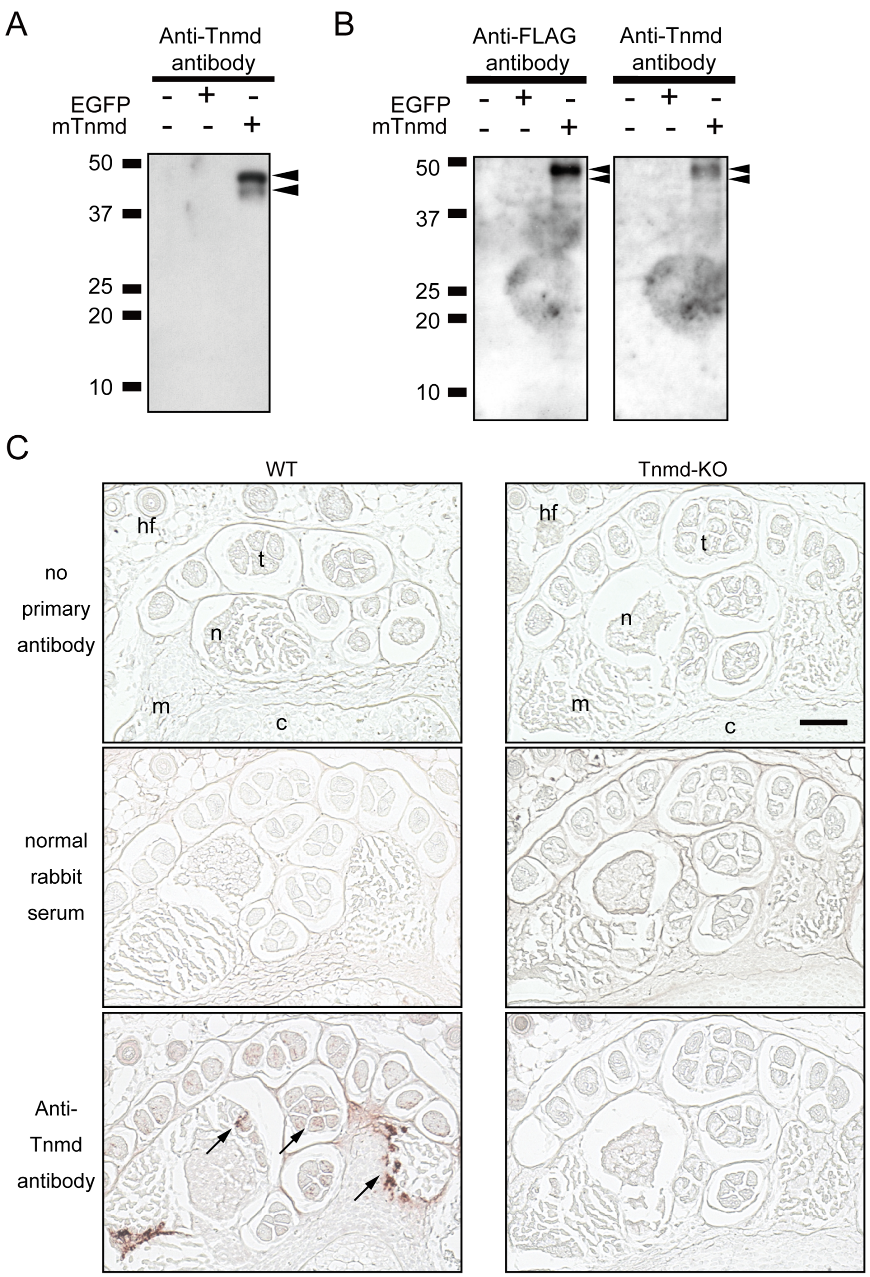

Supplemental Figure S1. Establishment of anti-Tnmd antibody.

(**A**) Detection of Tnmd transfected in NIH3T3 cells. Human Tnmd was transfected in NIH3T3 cells to evaluate antibody reactivity. (**B**) Detection of FLAG-tagged Tnmd transfected in NIH3T3 cells by western blot analysis. Cell lysates of transfected cells were first detected using the anti-FLAG M2 antibody, then subsequently with the anti-Tnmd antibody. (**C**) Immunohistological evaluation of anti-Tnmd antibody. WT and *Tnmd*-KO mice tail specimens were stained without the primary antibody, with normal rabbit serum, or with anti-Tnmd antibody. Arrow indicates the region of positive signal. c: cartilage, hf, hair follicle, m: muscle, n: nerve, t: tendon. Scale bar = 500 μm.
