# Supplementary material for: Tenomodulin Expression in the Periodontal Ligament Enhances Cellular Adhesion
Source: PLoS One. 2013 Apr 10;8(4):e60203. doi: 10.1371/journal.pone.0060203 (PMC3622668; doi:10.1371/journal.pone.0060203)
Supplement: Figure S2 — Histological findings of Tnmd –KO mouse teeth. H&E stainings of the maxilla first molar of 1, 2, 3, 4, and 6-week-old WT or Tnmd-KO mice are shown. Scale bar = 500 µm. Representative images are shown. (PDF) [file pone.0060203.s002.pdf]

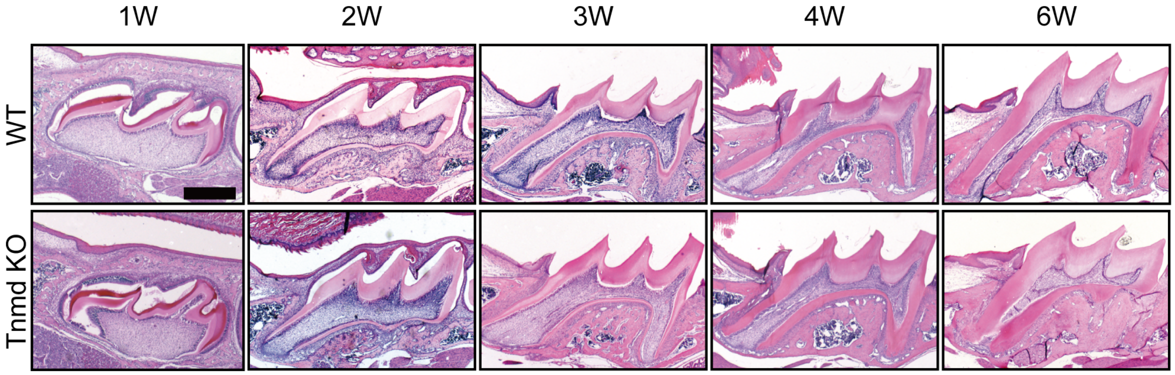

Supplemental Figure S2. Histological findings of *Tnmd*–KO mouse teeth.

H&E stainings of the maxilla first molar of 1, 2, 3, 4, and 6-week-old WT or *Tnmd*-KO mice are shown. Scale bar = 500 µm. Representative images are shown.
