# Supplementary material for: Tenomodulin Expression in the Periodontal Ligament Enhances Cellular Adhesion
Source: PLoS One. 2013 Apr 10;8(4):e60203. doi: 10.1371/journal.pone.0060203 (PMC3622668; doi:10.1371/journal.pone.0060203)
Supplement: Figure S3 — Micro CT analysis of craniofacial hard tissue in 10-week-old WT and Tnmd -KO mice. (A) Sagittal plane section image of WT and Tnmd-KO mice. Images are focused on a maxillary incisor, mandibular incisor or molar. (B) Frontal plane section image of WT and Tnmd-KO mice. Images are focused on a first (M1), second (M2), or third molar (M3) and on the temporomandibular joint (TMJ). (C) Horizontal plane section image of WT and Tnmd-KO mice. Images are focused on the plane, which intersects the molar root and external auditory canal. mxi: maxillary incisor; mdi: mandibular incisor; mx: maxilla; md: mandibule; tmj: temporomandibular joint; mxm: maxillary molar; mdm: mandibularmolar; eac: external auditory canal. (PDF) [file pone.0060203.s003.pdf]

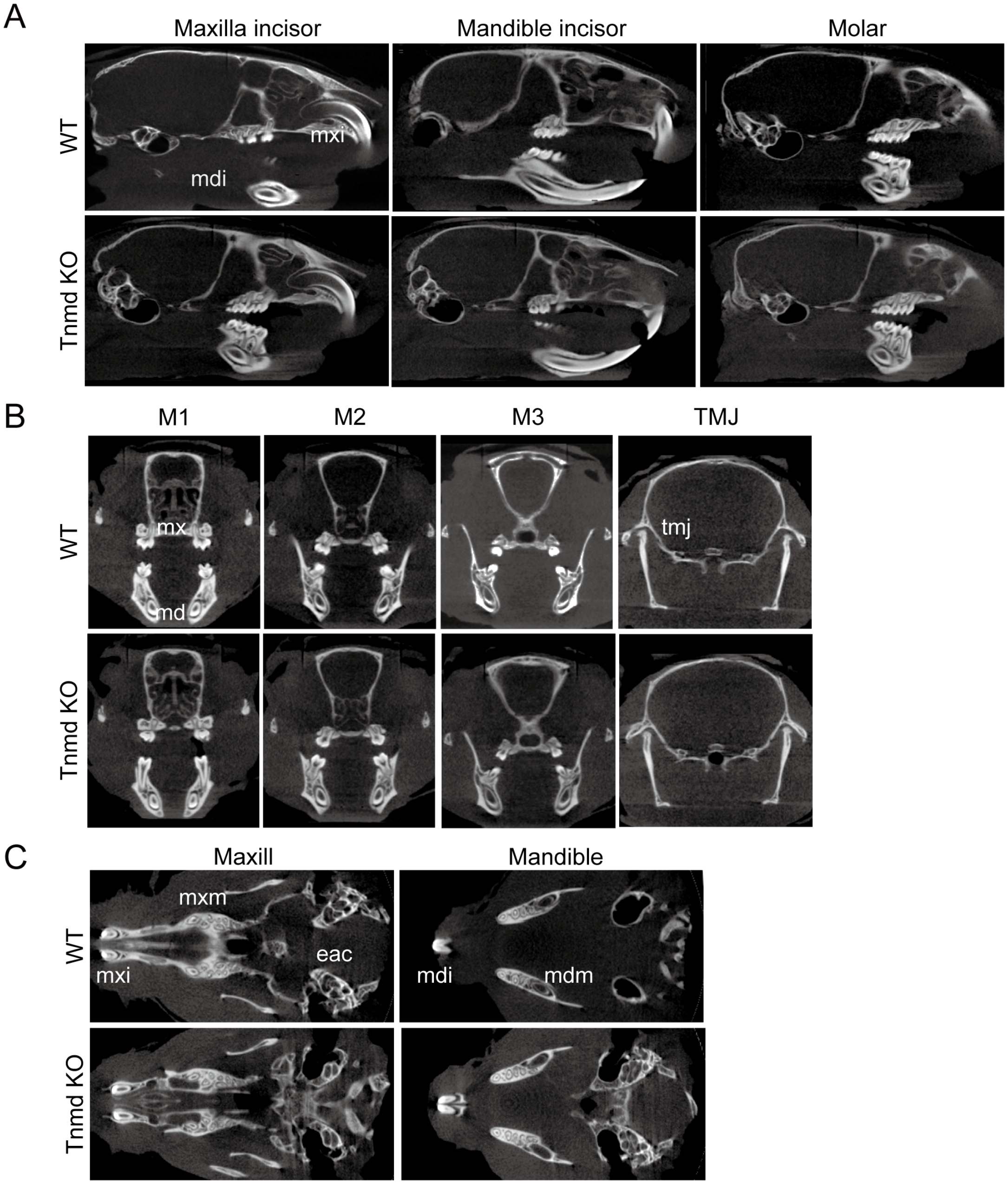

Supplemental Figure S3. Micro CT analysis of craniofacial hard tissue in 10-week-old WT and *Tnmd*-KO mice.

(**A**) Sagittal plane section image of WT and *Tnmd*-KO mice. Images are focused on a maxillary incisor, mandibular incisor or molar. (**B**) Frontal plane section image of WT and *Tnmd*-KO mice. Images are focused on a first (M1), second (M2), or third molar (M3) and on the temporomandibular joint (TMJ). (**C**) Horizontal plane section image of WT and *Tnmd*-KO mice. Images are focused on the plane, which intersects the molar root and external auditory canal. mxi: maxillary incisor; mdi: mandibular incisor; mx: maxilla; md: mandibule; tmj: temporomandibular joint; mxm: maxillary molar; mdm: mandibularmolar; eac: external auditory canal.
