# Supplementary material for: Tenomodulin Expression in the Periodontal Ligament Enhances Cellular Adhesion
Source: PLoS One. 2013 Apr 10;8(4):e60203. doi: 10.1371/journal.pone.0060203 (PMC3622668; doi:10.1371/journal.pone.0060203)
Supplement: Figure S4 — Examination of glycosylation in Tnmd by Tunicamycin treatment. Uncropped image of Figure 3B is shown. Full length Tnmd or EGFP is transfected to NIH3T3 cells. Tunicamycin was added 24 h after transfection, and cells were harvested at 48 h after transfection. No protein was detected lower than 37 kDa or higher than 50 kDa molecular weight. (PDF) [file pone.0060203.s004.pdf]

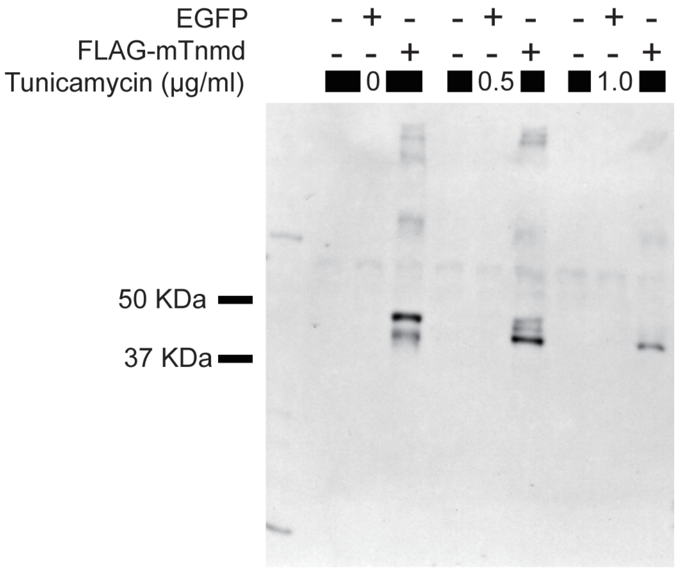

Supplemental Figure S4. Examination of glycosylation in Tnmd by Tunicamycin treatment.

Uncropped image of Figure 3B is shown. Full length Tnmd or EGFP is transfected to NIH3T3 cells. Tunicamycin was added 24 h after transfection, and cells were harvested at 48 h after transfection. No protein was detected lower than 37 kDa or higher than 50 kDa molecular weight.
