# Supplementary material for: Tenomodulin Expression in the Periodontal Ligament Enhances Cellular Adhesion
Source: PLoS One. 2013 Apr 10;8(4):e60203. doi: 10.1371/journal.pone.0060203 (PMC3622668; doi:10.1371/journal.pone.0060203)
Supplement: Figure S5 — Effect of Tnmd of Tnmd mutant transfection on cell adhesion. Cell adhesion assay to Fibronectin are shown. (A) Dose-dependent enhancement of cell adhesion by Tnmd. FLAG-tagged Tnmd was transfected in combination with pCAGGS-Venus in NIH3T3 cells. Cell adhesion to Fibronectin-coated culture dishes was determined. (B) Cell adhesion of WT and Tnmd-KO cells to Col I-coated culture dishes. (PDF) [file pone.0060203.s005.pdf]

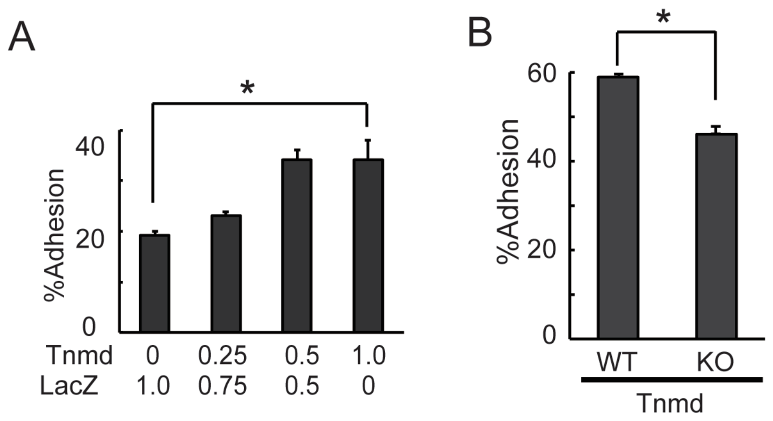

Figure S5. Effect of Tnmd of Tnmd mutant transfection on cell adhesion.

Cell adhesion assay to Fibronectin are shown. (**A**) Dose-dependent enhancement of cell adhesion by Tnmd. FLAG-tagged *Tnmd* was transfected in combination with pCAGGS-Venus in NIH3T3 cells. Cell adhesion to Fibronectin-coated culture dishes was determined. (**B**) Cell adhesion of WT and *Tnmd*-KO cells to Col I-coated culture dishes.
