# Supplementary material for: Tenomodulin Expression in the Periodontal Ligament Enhances Cellular Adhesion
Source: PLoS One. 2013 Apr 10;8(4):e60203. doi: 10.1371/journal.pone.0060203 (PMC3622668; doi:10.1371/journal.pone.0060203)
Supplement: Figure S6 — Subcellular localization of Tnmd in hPDL-TERT cells. The subcellular localization of the Tnmd protein in hPDL-TERT cells transfected with Tnmd was examined by ICC. NIH3T3 cells transfected with Tnmd were double stained with cell organelle markers and the anti-Tnmd . (A) Tnmd and plasma membrane. (B) Tnmd and Golgi apparatus. (C) Tnmd and β-Actin. (D) Tnmd and α-Tubulin. DAPI nuclear staining is shown in blue, cell organelle markers in green, and anti-Tnmd antibody in red. Scale bar = 50 µm. (PDF) [file pone.0060203.s006.pdf]

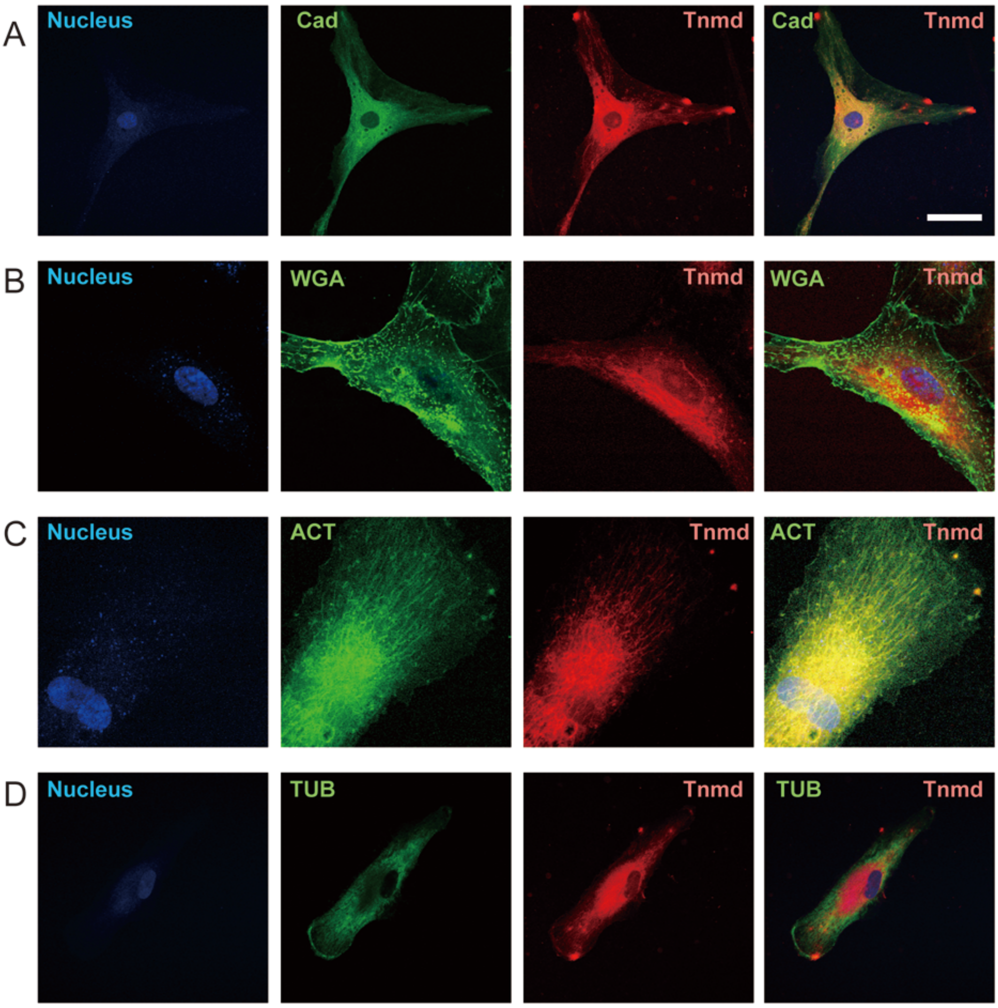

Figure S6. Subcellular localization of Tnmd in hPDL-TERT cells.

The subcellular localization of the Tnmd protein in hPDL-TERT cells transfected with Tnmd was examined by ICC. NIH3T3 cells transfected with Tnmd were double stained with cell organelle markers and the anti-Tnmd . (**A**) Tnmd and plasma membrane. (**B**) Tnmd and Golgi apparatus. (**C**) Tnmd and β-Actin. (**D**) Tnmd and α-Tubulin. DAPI nuclear staining is shown in blue, cell organelle markers in green, and anti-Tnmd antibody in red. Scale bar = 50 μm.
